# Reduction in cytokine production in colorectal cancer patients: association with stage and reversal by resection

**DOI:** 10.1054/bjoc.1999.1034

**Published:** 2000-02-01

**Authors:** A G Heriot, J B Marriott, S Cookson, D Kumar, A G Dalgleish

**Affiliations:** Colorectal Surgery Unit, Division of Oncology, St George's Hospital Medical School, Cranmer Terrace, Tooting, London, SW17 0RE, UK

**Keywords:** colorectal cancer, cytokines, immune suppression

## Abstract

The aim of this study was to assess monocyte/macrophage function, as defined by lipopolysaccharide (LPS)-induced production of tumour necrosis factor (TNF)-α, interleukin (IL)-10 and interferon (IFN)-γ by stimulated whole blood cultures in patients with colorectal carcinoma before and after surgical resection. Forty colorectal cancer patients prior to surgery and 31 healthy controls were studied. Heparinized venous blood was taken from colorectal cancer patients prior to surgery and from healthy controls. Serial samples were obtained at least 3–6 weeks post-operatively. Blood was stimulated with LPS for 24 h and supernatants were assayed for TNF-α, IFN-γ and IL-10 by enzyme-linked immunosorbent assay. LPS-induced production of TNF-α and of IFN-γ was reduced in patients with colorectal carcinoma compared to controls (TNF-α, 11 269 pg ml^−1^{12 598}; IFN-γ, 0.00 pg ml^−1^{226}; median {IQR}) (TNF-α, 20 576 pg ml^−1^{11 637}, *P*< 0.0001; IFN-γ, 1048 {2428}, *P* = 0.0051, Mann–Whitney *U* -test). Production in patients after surgery had increased (TNF-α: 17 620 pg ml^−1^{7986}; IFN-γ: 410 pg ml^−1^{2696}; mean {s.d.}) and were no longer significantly reduced when compared to controls (TNF-α, *P* = 0.28; IFN-γ, *P* = 0.76). Production of TNF-α and IFN-γ prior to surgery were reduced to a greater extent in patients with Dukes' stage C tumours compared to those with Dukes' stage A and B stage. There was no difference in IL-10 production between any group. Monocytes/macrophages from patients with colorectal carcinoma are refractory to LPS stimulation as reflected by reduction in TNF-α and IFN-γ production and this is more pronounced in patients with advanced stage tumours. This suppression is not mediated by IL-10 and disappears following surgical resection of the tumour. This provides evidence for tumour induced suppression of immune function in patients with colorectal cancer and identifies a potential therapeutic avenue. © 2000 Cancer Research Campaign


*British Journal of Cancer* (2000) **82**(5), 1009–1012
© 2000 Cancer Research Campaign
DOI: 10.1054/ bjoc.1999.1034, available online at http://www.idealibrary.com on IDEAL®

### Reduction in cytokine production in colorectal cancer patients: association with stage and reversal by resection

**AG Heriot[1]*, JB Marriott[2]*, S Cookson[2], D Kumar[1] and AG Dalgleish[2]**

[1]Colorectal Surgery Unit and [2]Division of Oncology, St George's Hospital Medical School, Cranmer Terrace, Tooting, London SW17 0RE, UK

**Summary** The aim of this study was to assess monocyte/macrophage function, as defined by lipopolysaccharide (LPS)-induced production of tumour necrosis factor (TNF)-α, interleukin (IL)-10 and interferon (IFN)-γ by stimulated whole blood cultures in patients with colorectal carcinoma before and after surgical resection. Forty colorectal cancer patients prior to surgery and 31 healthy controls were studied. Heparinized venous blood was taken from colorectal cancer patients prior to surgery and from healthy controls. Serial samples were obtained at least 3–6 weeks post-operatively. Blood was stimulated with LPS for 24 h and supernatants were assayed for TNF-α, IFN-γ and IL-10 by enzyme-linked immunosorbent assay. LPS-induced production of TNF-α and of IFN-γ was reduced in patients with colorectal carcinoma compared to controls (TNF-α, 11 269 pg ml$^{-1}$ {12 598}; IFN-γ, 0.00 pg ml$^{-1}$ {226}; median {IQR}) (TNF-α, 20 576 pg ml$^{-1}$ {11 637}, $P < 0.0001$; IFN-γ, 1048 {2428}, $P = 0.0051$, Mann–Whitney *U*-test). Production in patients after surgery had increased (TNF-α: 17 620 pg ml$^{-1}$ {7986}; IFN-γ: 410 pg ml$^{-1}$ {2696}; mean {s.d.}) and were no longer significantly reduced when compared to controls (TNF-α, $P = 0.28$; IFN-γ, $P = 0.76$). Production of TNF-α and IFN-γ prior to surgery were reduced to a greater extent in patients with Dukes' stage C tumours compared to those with Dukes' stage A and B stage. There was no difference in IL-10 production between any group. Monocytes/macrophages from patients with colorectal carcinoma are refractory to LPS stimulation as reflected by reduction in TNF-α and IFN-γ production and this is more pronounced in patients with advanced stage tumours. This suppression is not mediated by IL-10 and disappears following surgical resection of the tumour. This provides evidence for tumour induced suppression of immune function in patients with colorectal cancer and identifies a potential therapeutic avenue. © 2000 Cancer Research Campaign

**Keywords:** colorectal cancer; cytokines; immune suppression

In spite of a perceived shortage of evidence to support Burnett's immune surveillance theory for the majority of solid tumours (for example, the relative absence of non-viral tumours in AIDS patients) there is considerable interest in the hypothesis that the immune system may be actively suppressed in patients with malignant disease. Patients with solid tumours have been shown to have reduced cell-mediated immunity as assessed by skin testing (Medical Oncology Society, 1979; King et al, 1997). Furthermore, it has been reported that the risk of tumour recurrence is increased if this immune suppression is not reversed (Cole and Humphrey, 1985). A reduction in the number of peripheral blood T-lymphocytes and in the proportions of CD4 and CD8 T-lymphocytes in patients with solid tumours (Tancini et al, 1990; Tsutsui et al, 1992) and a reduced CD4 lymphocyte count occur in patients with advanced colorectal carcinoma when compared to normal controls (Arista et al, 1994). Lymphocyte numbers and proportions may give an indication of immune status but fail to give an absolute measure of tumour-specific immune function, although the greater reduction in lymphocyte numbers in patients with advanced tumours, compared to patients with less advanced tumours, suggests an associative role.

*Received 24 May 1999*
*Revised 27 August 1999*
*Accepted 14 September 1999*

*Correspondence to:* AG Dalgleish

Cytokine production by stimulated monocytes/macrophages during antigen presentation is an integral part of the immune response and involves production of both pro-inflammatory cytokines such as tumour necrosis factor-α (TNF-α) and interferon-γ (IFN-γ) and immunosuppressive cytokines such as interleukin-10 (IL-10). The aim of this study was to investigate the effect of primary colorectal carcinoma on monocyte/macrophage function, as represented by the production of TNF-α, IFN-γ and IL-10 in stimulated whole blood cultures, both prior to and following surgical resection of the tumour.

### MATERIALS AND METHODS

Heparinized venous blood was collected from 40 patients (26 male, 14 female) with primary colorectal cancer prior to treatment and 31 similarly aged controls (14 male, 17 female). All patients were informed of the study and gave written consent. The study was approved by the local ethical committee. Serial blood samples were obtained following surgical resection of the tumour, if performed, and at outpatient visits during post-operative follow-up. Clinical data regarding the patient and tumour histology were recorded. In the majority of the patients, the white blood count and

This work has been presented as an abstract at the British Society of Gastroenterology, Harrogate, March 1998, and the Digestive Diseases Week, New Orleans, May 1998.

*These authors contributed equally to this study.

differential was also measured prior to treatment. All controls were either healthy or had benign, non-inflammatory conditions and none were taking steroids or any form of immunosuppression.

Blood was diluted in RPMI-1640 medium (1:4) + glutamine (2 mM) and either stimulated with lipopolysaccharide (LPS; 1 μg $ml^{-1}$; Sigma, *Escherichia coli* serotype 0127:B8) by incubation in 24-well plates at 37°C/5% carbon dioxide for 24 h or left unstimulated under the same conditions. Cell-free supernatants were collected by microcentrifugation and stored in aliquots at –70°C until analysis. Supernatants were assayed for TNF-α, IFN-γ and IL-10 by enzyme-linked immunosorbent assay (ELISA) using an assay procedure and reagents (anti-cytokine capture monoclonal antibody, biotinylated anti-cytokine detecting antibody and recombinant cytokine) provided by Pharmingen (Cambridge Bioscience, UK). In each case the manufacturers' instructions were followed exactly.

### Statistical analysis

Data are presented as mean ± standard deviation (s.d.) from the mean. Statistical analyses were performed using Mann–Whitney *U*-tests for unpaired two group non-parametric data and Kruskal–Wallis tests for non-parametric data when comparing more than two groups. Analysis of variance, followed by Student's *t*-test, were used to compare age. In all tests, significance was accepted at $P < 0.05$.

### RESULTS

No cytokine production was observed in unstimulated cultures and all the results presented below represent the results from LPS-stimulated cultures only.

Levels of TNF-α, IFN-γ and IL-10 were measured in culture supernatants derived from patients prior to treatment. Production of TNF-α in patients with colorectal tumours (median, 11 269 pg $ml^{-1}$, Interquartile range [IQR] 12 598 pg $ml^{-1}$, $n = 37$) was significantly reduced compared to that of the control patients (20 576 pg $ml^{-1}$, 11 637 pg $ml^{-1}$, $n = 31$; $P < 0.0001$) (Figure 1A). Production of IFN-γ in patients with colorectal tumours (median, 0.00 pg $ml^{-1}$, IQR 226 pg $ml^{-1}$, $n = 40$) was also significantly reduced when compared to controls (1048 pg $ml^{-1}$, 2428 pg $ml^{-1}$, $n = 31$; $P = 0.0051$) (Figure 1B). There was no difference in the level of IL-10 between patients with colorectal tumours ($O.D._{450}$: median 0.406, IQR 0.204; $n = 38$) and controls ($O.D._{450}$: median 0.436, IQR 0.048, $n = 24$; $P$ = NS) (Figure 1C). There was no significant difference in age between the patients with colorectal tumours (70.5 years ± 11.4 years) and the control patients (66.2 years ± 10.5 years; $P$ = NS). There was no difference between patients with colorectal cancer and controls in white blood count ($8.7 \times 10^9$ $l^{-1}$ ± 3.1, $n = 33$ vs $7.6 \times 10^9$ $l^{-1}$ ± 1.8, $n = 26$ respectively; $P$ = NS), lymphocyte count ($1.62 \times 10^9$ $l^{-1}$ ± 0.6, $n = 33$ vs $1.9 \times 10^9$ $l^{-1}$ ± 0.74, $n = 26$ respectively; $P$ = NS), or monocyte count ($0.7 \times 10^9$ $l^{-1}$ ± 0.25, $n = 33$ vs $0.6 \times 10^9$ $l^{-1}$ ± 0.27, $n = 26$ respectively; $P$ = NS), and there was no difference across Dukes' stages.

LPS-induced production of TNF-α and IFN-γ and IL-10 were assayed in all patients who were at least 20 weeks post-surgery (range 20–44 weeks) with the most recent sample used. Production of TNF-α in patients with colorectal cancer had increased compared to production prior to resection of the tumour (median 17 620 pg $ml^{-1}$, IQR 7986 pg $ml^{-1}$, $n = 15$) and was no longer significantly less than the control patients ($P = 0.28$) (Figure 1A). Production of IFN-γ in the cancer patients (median 410 pg $ml^{-1}$, IQR 2696 pg $ml^{-1}$, $n = 11$) was also increased compared to prior to

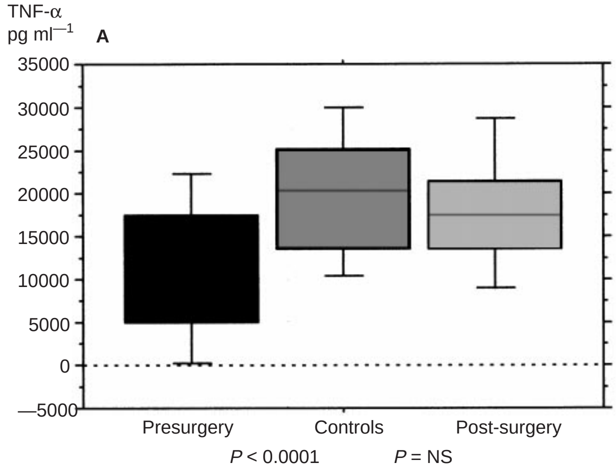

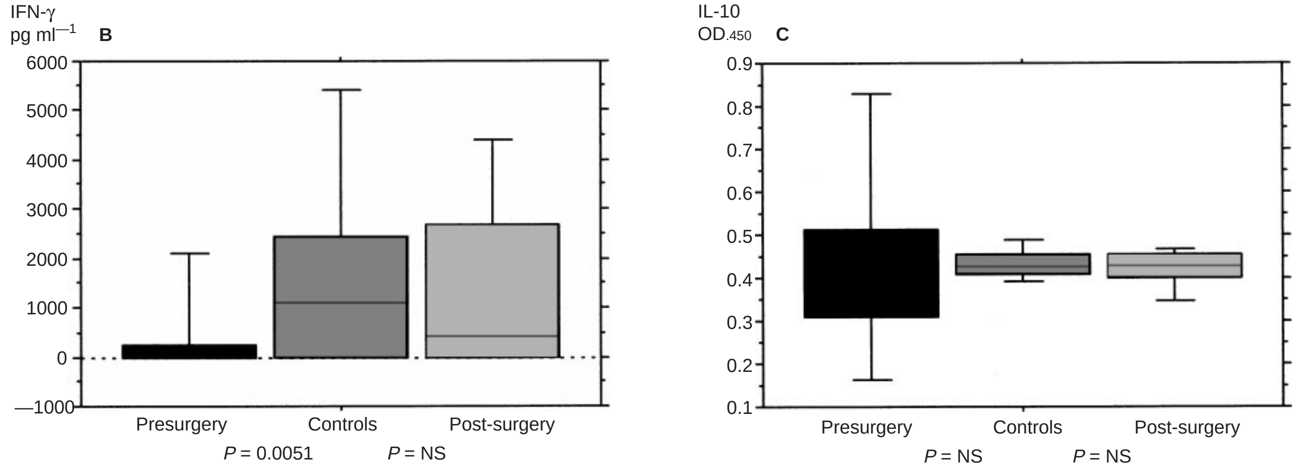

**Figure 1** (**A**) TNF-α levels in stimulated whole blood in patients with colorectal cancer, prior to, and post-surgical resection, versus controls. (**B**) IFN-γ levels in stimulated whole blood in patients with colorectal cancer, prior to, and post-surgical resection, versus controls. (**C**) IL-10 levels in stimulated whole blood in patients with colorectal cancer, prior to, and post-surgical resection, versus controls. NS: not significant

 *© 2000 Cancer Research Campaign*

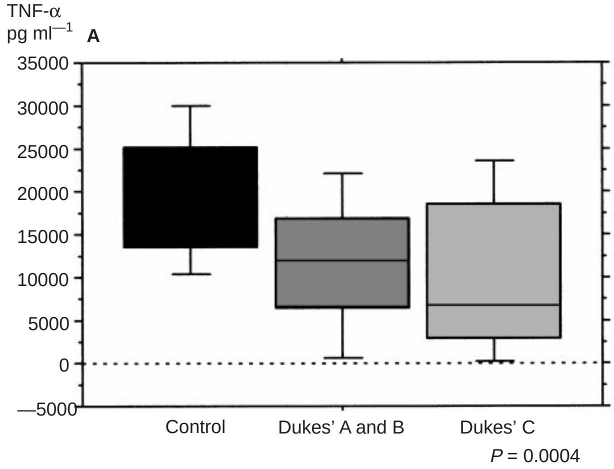

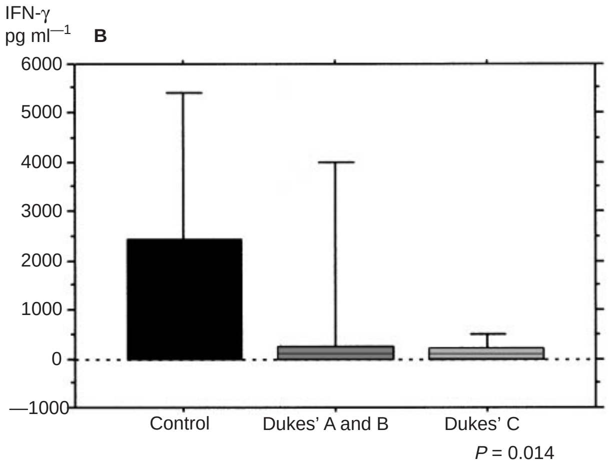

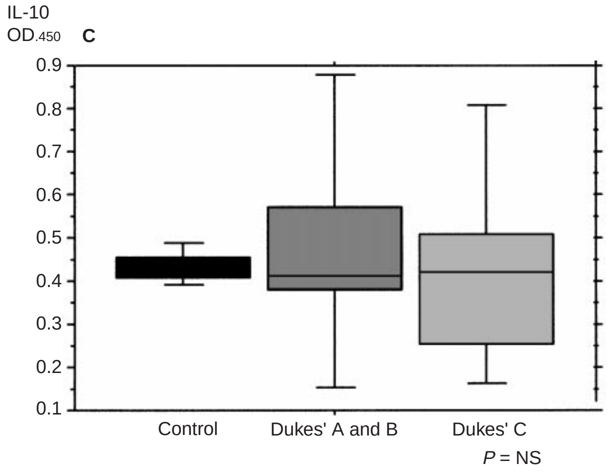

**Figure 2** (**A**)TNF-α levels in stimulated whole blood in patients with colorectal cancer, divided by tumour stage (Duke’s stage), versus controls. (**B**) IFN-γ levels in stimulated whole blood in patients with colorectal cancer, divided by tumour stage (Duke’s stage), versus controls. (**C**) IL-10 levels in stimulated whole blood in patients with colorectal cancer, divided by tumour stage (Duke’s stage), versus controls. NS: not significant surgery and was no longer significantly less than the control patients ($P = 0.76$) (Figure 1B). The level of IL-10 was unchanged (median 0.423, IQR 0.053, $n = 16$) and remained similar to the control patients (Figure 1C).

Levels of TNF-α, IFN-γ and IL-10 production prior to any treatment were compared to the Dukes’ stage of the tumour. Due to the limited number of Dukes’ A stage tumours, Dukes’ A and B tumour stages were combined, analysed as a single group of tumours with spread confined to the bowel wall and compared with Dukes’ C tumours, with spread to lymph nodes, and with controls. There was a significant reduction in TNF-α levels in the cancer patients compared to controls (median 20 576 pg ml$^{-1}$, IQR 11 637 pg ml$^{-1}$, $n = 31$) and this reduction was greater in patients with Dukes’ C stage tumours (median 6894 pg ml$^{-1}$, IQR 15 687 pg ml$^{-1}$, $n = 19$) than with those with Dukes’ A or B stage tumours (median 11 808 pg ml$^{-1}$, IQR 10 352 pg ml$^{-1}$, $n = 21$; $P = 0.0004$) (Figure 2A). There was a significant reduction in IFN-γ levels in the cancer patients compared to controls (median 1048 pg ml$^{-1}$, IQR 2428 pg ml$^{-1}$, $n = 31$) and this reduction was greater in patients with Dukes’ C stage tumours (median 0.00 pg ml$^{-1}$, IQR 213 pg ml$^{-1}$, $n = 19$) than with those with Dukes’ A or B stage tumours (median 13.0 pg ml$^{-1}$, IQR 247 pg ml$^{-1}$, $n = 21$; $P = 0.014$) (Figure 2B). There was no demonstrable difference in IL-10 production in the cancer patients compared to the controls (median 0.436, IQR 0.048, $n = 24$) in either Dukes’ stage A and B tumours (median 0.406, IQR 0.191, $n = 18$) or Dukes’ stage C tumours (median 0.422, IQR 0.255, $n = 19$; $P = 0.922$) and there was no difference in IL-10 levels across the Dukes’ stages (Figure 2C).

### DISCUSSION

The interaction between the host immune system and malignant tumours is complex and has been the subject of much debate. Only melanoma, renal cell carcinoma and perhaps prostate carcinoma (Hrouda et al, 1997) have clearly been accepted as immunologically sensitive solid tumours; immunotherapies, such as treatment with IFN-α, IL-2 and vaccination strategies have provided some evidence of efficacy (Mittelman et al, 1990). However, a large number of patients do not respond to these therapies and such approaches are mostly not envisaged with other tumours. One of the many ways that a solid tumour can evade an effective immune response is to induce immunosuppressive cytokines, such as IL-10, which inhibit antigen presenting function (and thus cell-mediated immunity), via down-regulation of MHC class II expression. Therefore, the measurement of cytokine production by stimulated monocytes provides an insight into the ability of the immune system to respond to external stimuli.

The hypothesis of tumour-mediated immune suppression, as suggested by previous studies assessing cell-mediated immunity by skin hypersensitivity (Medical Oncology Society, 1979; King et al, 1997) is supported by our study. We have shown a highly significant reduction in production of TNF-α and IFN-γ by LPS-stimulated whole blood cultures derived from patients with colorectal cancer as compared to similarly aged controls. This suppression of cytokine production appears to be selective since

*© 2000 Cancer Research Campaign*

the production of IL-10, a cytokine responsible for monocyte deactivation, was not affected, and as there was no difference in leucocyte count between cancer patients and controls, it suggests a functional suppression rather than a reduction in leucocyte numbers as has been demonstrated with advanced colorectal tumours (Arista et al, 1994). Elsasser-Beile et al (1992) reported similar results with a reduction in production of TNF-α and IFN-γ in patients with colorectal carcinoma and no change in leucocyte count though this was only studied at a single time point and the effect of resection was not assessed.

Interestingly, we have also shown that suppression of TNF-α and IFN-γ production appears to disappear following resection of the tumour. Surgery is known to result in a transient immune suppression which usually resolves after 6–9 days (Hammer et al, 1992). However, in this study samples were taken from patients prior to surgery and (after tumour resection) at a time distant (greater than 20 weeks) to allow immunosuppression from the surgery itself to resolve. McMillan et al (1997) have previously reported that impaired immunity, as measured by decreased lymphocyte subset populations, is important in tumour recurrence in colorectal cancer. However, at present the follow-up on the patients in this study is too limited to assess whether this is also the case for cytokine production. It is also interesting to note that impaired monocyte/macrophage function is apparent in patients with primary tumours and is not restricted to patients with advanced or metastatic disease as has previously been shown (Arista et al, 1994; King et al, 1997). Our data strongly suggest that the degree of functional impairment increases with tumour stage. This may be due to specific characteristics of the tumour which results in an increased propensity for invasion.

TNF-α production may also be an indicator of the degree of tumour cell lysis during antibody-dependent cell-mediated cytotoxicity as it is released by monocytes when cross-linked to tumour cells by anti-tumour antibody (Pullyblank et al, 1991). Furthermore, tumour-induced down-regulation of IFN-γ production may represent a mechanism of inhibiting tumour-specific CTL. IFN-γ has an important role in the regulation of tumour cell lysis and has been shown to increase the sensitivity of colon carcinoma cell lines to Fas-mediated apoptosis (Darcy et al, 1998), possibly via the induction of caspase expression. IFN-γ is also crucial for the induction of IL-12 which is a powerful inducer of Th-1 type immunity and an important cytokine in the generation of anti-tumour immunity. Interestingly, a recent study by O’Hara et al (1998) has demonstrated that the production of IL-12 is deficient in patients with colorectal cancer.

There is now considerable evidence that colorectal cancer will respond to immunological interventions. The use of IL-12, either as a preoperative treatment or in metastatic colorectal cancer is claimed to be associated with an improved survival (compared to current best supportive care) (Barni et al, 1995; Brivio et al, 1996). Reithmuller and colleagues reported that a monoclonal antibody (17-1A) to a colorectal antigen resulted in an improved survival for patients with Dukes’ stage C colon cancer when given post resection (Reithmuller et al, 1994).

In conclusion, our study suggests the need for therapeutic strategies for both early and metastatic colorectal cancer to include immunotherapy in order to boost cell mediated immunity and monocyte function. This may also be required to allow other passive or active immunotherapies to be effective. Further studies should include prospective monitoring of patients with poor cytokine responses given standard treatments with or without cell-mediated enhancement, such as IL-2 administration. If relatively non-toxic immunotherapy can prolong the survival of patients with Dukes’ C (and hence probably Dukes’ B) colorectal cancer it may prevent the need for exposure to 5-fluorouracil, with its associated side-effects, which remains the current standard chemotherapy.

### REFERENCES

Arista M, Callopoli A, De Franceschi L, Santini A, Schiratti M, Di Filippo F and Gandolfo GM (1994) Flow cytometric study of lymphocyte subsets in patients at different stage of colorectal carcinoma. *Dis Colon Rectum* **37**: S30–4

Barni S, Lissoni P, Cazzaniga M, Ardizzoia A, Meregalli S, Fossati V, Fumagalli L, Brivio F and Tancini G (1995) A randomized study of low-dose subcutaneous interleukin-2 plus melatonin versus supportive care alone in metastatic colorectal cancer patients progressing under 5-fluorouracil and folates. *Oncology* **52**: 243–245

Brivio F, Lissoni P, Alderi G, Barni S, Lavorato F and Fumagalli L (1996) Preoperative interleukin-2 subcutaneous immunotherapy may prolong the survival time in advanced colorectal cancer patients. *Oncology* **53**: 263–268

Cole W and Humphrey L (1985) Need for immunologic stimulators during immunosuppression produced by major cancer surgery. *Ann Surg* **202**: 9–20

Darcy P, Kershaw M, Trapani J and Smyth M (1998) Expression in cytotoxic T lymphocytes of a single chain anti-carcinoembryonic antigen antibody. Redirected Fas ligand-mediated lysis of colon carcinoma. *Eur J Immunol* **28**: 1663–1672

Elsasser Beile U, von Kleist S, Fischer R and Monting JS (1992) Impaired cytokine production in whole blood cell cultures from patients with colorectal carcinomas as compared to benign colorectal tumors and controls. *J Clin Lab Anal* **6**: 311–314

Hammer J, Nielsen H, Moesgaard F and Kehlet H (1992) Duration of postoperative immunosuppression assessed by repeated delayed type hypersensitivity skin tests. *Eur Surg Res* **24**: 133–137

Hrouda D, Muir G and Dalgleish A (1997) The role of immunotherapy for urological tumours. *Br J Urol* **79**: 307–316

King J, Caplehorn J, Ross W and Morris D (1997) High serum carcinembryonic antigen concentration in patients with colorectal liver metastases is associated with poor cell-mediated immunity, which is predictive of survival. *Br J Surg* **84**: 1382–1385

McMillan DC, Fyffe GD, Wotherspoon HA, Cooke TG and McArdle CS (1997) Prospective study of circulating T-lymphocyte subpopulations and disease progression in colorectal cancer. *Dis Colon Rectum* **40**: 1068–1071

Medical Oncology Society (1979) Delayed cutaneous hypersensitivity (DCH) reactions in normal populations by multitest; effect of sex and age. *5th Annual Meeting of the Medical Oncology Society*, 1–3 December, Nice, France. Springer: Berlin

Mittelman A, Huberman M, Puccio C, Fallon B, Tessitore J, Savona S, Eyre R, Gafney E, Wick M, Skelos A, et al (1990) A phase I study of recombinant human interleukin-2 and alpha-interferon-2a in patients with renal cell cancer, colorectal cancer and malignant melanoma. *Cancer* **66**: 664–669

O’Hara R, Greenman J, Drew P, McDonald AW, Duthie GS, Lee PW and Monson JR (1998) Impaired interleukin-12 production is associated with a defective anti-tumor response in colorectal cancer. *Dis Colon Rectum* **41**: 460–463

Pullyblank A, Guillou P and Monson J (1991) Interleukin 1 and tumour necrosis factor alpha may be responsible for the lytic mechanism during anti-tumour antibody dependent cell-mediated cytotoxity. *Br J Cancer* **72**: 601–606

Riethmuller G, Schneider-Gadicke E, Schlimok G, Schmiegel W, Raab R, Hoffken K, Gruber R, Pichlmaier H, Hirche H, Pichlmayr R, et al (1994) Randomised trial of monoclonal antibody for adjuvant therapy of resected Dukes’ C colorectal carcinoma. German Cancer Aid 17-1A Study Group [see comments]. *Lancet* **343**: 1177–1183

Tancini G, Barni S, Rescaldani R, Fiorelli G, Viviani S and Lissoni P (1990) Analysis of T helper and suppressor lymphocyte subsets in relation to the clinical stage of solid neoplasms. *Oncology* **47**: 381–384.

Tsutsui S, Morita M, Kuwano H, Matsuda H, Mori M, Okamura S and Sugimachi K (1992) Influence of preoperative treatment and surgical operation on immune function of patients with oesophageal carcinoma. *J Surg Oncol* **49**: 176–181

*© 2000 Cancer Research Campaign*
